# Work-related stress and future sick leave in a working population seeking care at primary health care centres: a prospective longitudinal study using the WSQ

**DOI:** 10.1186/s12889-022-13269-8

**Published:** 2022-04-28

**Authors:** Anna-Maria Hultén, Pernilla Bjerkeli, Kristina Holmgren

**Affiliations:** 1 Unit of Occupational Therapy, Department of Health and Rehabilitation, Institute of Neuroscience and Physiology, Sahlgrenska Academy, University of Gothenburg, Gothenburg, Sweden; 2 Department for Public Health Research, University of Skövde, Box 408, 541 28 Skövde, Sweden

**Keywords:** Organisational climate, Primary health care, Psychosocial risk factors, Sickness absence, Work commitment, Workers’ health, Work Stress Questionnaire (WSQ)

## Abstract

**Background:**

Studying the relationship between work-related stress and sick leave is valuable in identifying and assessing employees at risk of sick leave, but also in developing interventions and taking actions for workers’ health. The overall aim of this study was to analyse the association between work-related stress, measured with the work stress questionnaire (WSQ), and registered sick leave in a working population seeking care at primary health care centres in Sweden.

**Methods:**

A prospective longitudinal study was performed with 232 employed patients aged 18–64 years seeking care for mental and/or physical health complaints at seven primary health care centres. Bivariate logistic regression analysis adjusted for educational level, occupational class and marital status was performed using questionnaire data on work-related stress and sociodemographic factors collected between May 2015 until January 2016 together with registered sick leave data from a national database.

**Results:**

High stress due to indistinct organization and conflicts was reported by 21% (*n* = 49), while 45% (*n* = 105) reported high stress due to individual demands and commitment. Thirty-six percent were on sick leave for 15 days or more during 12 months after baseline. The odds of being on registered sick leave during this period was approximately twice as high for patients perceiving high stress due to indistinct organization and conflicts (OR 2.25, 95% CI 1.18;4.26), high stress due to individual demands and commitment (OR 2.21, 95% CI 1.28;3.82), low influence at work (OR 2.07, 95% CI 1.20;3.57), or high interference between work and leisure time (OR 2.19, 95% CI 1.27;3.80). Perceiving high stress due to both indistinct organization and conflicts as well as individual demands and commitment quadrupled the odds of sick leave, OR 4.15 (95% CI 1.84; 9.38).

**Conclusions:**

Work-related stress and sick leave were prevalent among the patients. Perceiving one or more of the work-related stressors and stress increased the odds of registered sick leave between two to four times. Hence, to capture the dynamic interaction between the individual and the work environment, a wide spectrum of factors must be considered. In addition, primary health care could be a suitable arena for preventing sick leave due to work-related stress.

**Trial registration:**

ClinicalTrials.gov. Identifier: NCT02480855. Registered 20 May 2015.

**Supplementary Information:**

The online version contains supplementary material available at 10.1186/s12889-022-13269-8.

## Background

The health and well-being of working age individuals is strongly affected by factors related to work [1]. For decades, the physical environment and occupational safety were in focus. As work tasks, labour standards and the innate meaning of work have changed in the European Union, the mental health at work places and thereby the social and organizational work environment has become more important for the promotion of health and the prevention of ill health [2, 3]. Apart from increasing mental and emotional work demands such as high motivation and creativity [4], changes are for example seen in flexible work, mobility and the composition of the labour market [3]. In addition, unforeseen and rapid changes in the labour market and the working conditions have developed in times of crisis [5]. However, the variation in working conditions and perceived health thereof is large. In Europe, structural inequalities and differences regarding gender, foreign origin, employment status and occupation are still important for job quality and working conditions [6]. Hence, individuals, work places and sectors are different and the health of the working population is therefore affected in various ways. The adverse and numerous negative health effects of work, and especially work-related stress, is a major cause for sick leave and thereby missed income, productivity losses and health care costs [7, 8]. It is therefore important to understand and quantify the association between work-related stress and sick leave.

Extensive research has been performed to understand the relationship between stressors or stress and ill health. However, the health effects are complex, since stress affects multiple bodily systems leading to mental, physical and behavioural ill health [9–11]. Overarching health measures, such as sick leave measures, have therefore been used to quantify the perceived ill health. Different general psychosocial risk factors, such as demands, job resources and justice [12–15], are associated with sick leave. In addition, the degree of and imbalance between stress inducing and stress preventing psychosocial risk factors at work, have been used to conceptualize the risk of employees’ work-related stress [13, 14, 16]. Moreover, the interdependence between factors external to and internal to work has also been emphasized [17]. However, decreasing the negative impact of these generic factors is not sufficient to prevent ill health due to work-related stress, since there is an individual variability to the same organizational and social environment [18]. Therefore, both the needs, values and abilities of the individual and the demands, goals and resources inherent in the specific occupational context have to be addressed, to understand the work-related stress mechanisms and find useful measures [19].

General conceptual models of work-related stress and related self-assessment questionnaires focus on those environmental and/or personal characteristics that are considered most important to explain the emergence of stress and any direct or indirect health effects thereof [14, 16, 20]. This study is founded on the transactional perspective on occupation [21]. Thereby, neither the individual nor the experienced environment is prioritized in thinking about stress reactions or behaviour. Instead, attention is directed to the aspects of experience that are important in a given situation in time [21]. For example, perceiving the degree of influence or the quality of the organisation of work as low has been found to significantly increase the risk of sick leave [22–24]. In addition, there is evidence that the work situation can interfere with time spent with family and friends to a degree that it affects sick leave [17]. Further, the individual’s approach to work is of relevance. Research has shown that the degree of engagement and commitment to work are important explanatory factors for sick leave [14, 25]. Holmgren et al. [26] found that person-related factors such as placing high demands on oneself, taking more responsibility than expected and having difficulty in setting limits contributed to ill health and sick listing. Research has also shown that both being strongly committed to work and perceiving the quality of the organisation of work as low increased the risk of future sick leave [24, 25]. In addition, other studies have shown a relatedness between organisational aspects of work and commitment [27, 28].

Sociodemographic background factors of the individual worker, such as age, gender, place of residence, life-style and socio-economic status, are important risk factors for sick leave independent of diagnosis or underlying disease [12]. Since these background factors also affect the degree of perceived work-related stress [29], Kivimäki et al. [5] emphasize the need to consider the explanatory effects of such factors for the relationship between stress and sick leave. Research has shown that among other things gender, social position and age can affect the association between occupational/ job characteristics, work-related stress and sick leave, even if the causal pathways were not established [30–32].

In Sweden, general practitioners at primary health care centres regularly issue medical certificates, as an important basis for decisions on sick leave [33]. In addition, work-related ill health is a common cause for seeking care [34] and symptoms of burnout and exhaustion are prevalent among primary health care patients [35]. Moreover, people consult the primary health care for different physical and mental symptoms long before realizing that work-related stress could be the underlying cause [36]. Altogether, these circumstances make primary health care an important stakeholder for disease prevention and health promotion to reduce sick leave due to work-related stress. Studying the relationship between work-related stress and sick leave in this context is valuable, since it could facilitate the identification of persons at risk of sick leave due to work-related stress, the development of health interventions and the coordination of relevant actions.

The overall aim of this study was to analyse the association between self-assessed work-related stress and registered sick leave, that is sick leave spells lasting more than 14 days, in a working population seeking care at primary health care centres in South west Sweden. The specific aims were (a) to describe the distribution of self-assessed work-related stress and registered sick leave among women and men and (b) to investigate the association between self-assessed work-related stress at baseline and registered sick leave for a period from baseline and 12 months onwards.

## Methods

A prospective longitudinal study was designed to analyse the association between self-assessed work-related stress, as measured with the Work Stress Questionnaire (WSQ), and registered sick leave in a population of patients seeking care at primary health care centres in the Västra Götaland region in Sweden.

### Setting and study population

Baseline questionnaire data from a randomized controlled trial (RCT) of the TIDAS-project was used for the analysis. Ethics approval was provided by the Regional Ethical Review Board in Gothenburg, Sweden. The two-armed parallel trial has previously been described in detail in a study protocol [37]. The trial was set to evaluate the effect of a brief intervention, where the WSQ was used combined with feedback at consultation with a general practitioner, on patients’ sick leave. Altogether, 63 general practitioners and 271 patients at seven primary health care centres were included.

The trial targeted non-sick-listed employed women and men aged 18 to 64 years who sought care for depression, anxiety, musculoskeletal disorders, gastro-intestinal, cardiovascular conditions or other potentially stress related symptoms. Patients currently receiving sickness benefits and patients with seven days of sick leave or more the last month were excluded, as were pregnant women due to the risk for pregnancy-related sick leave during the follow-up period. In addition, patients seeking care for diabetes, urinary tract infections, infections (e.g. cold, sore throat), chronic obstructive lung disease, fractures, lump and spots, allergy, psychiatric diagnoses (e.g. schizophrenia and bipolar disorder), prolonging of sick leave as well as medical check-ups were excluded. Patients who fulfilled the criteria were consecutively invited to participate in the trial. A research assistant on site coordinated the identification and invitation of eligible patients, gave oral and written information about the study as well as obtained informed consent for the study.

The present study included patients who participated in the RCT and completed their assessment of work-related stress with the WSQ.

### Data sources

Data concerning work-related stress, assessed by the WSQ, and sociodemographic data were collected using a self-administered questionnaire at baseline between May 2015 until January 2016. Patients in the intervention group completed the self-administered questionnaire in the waiting room before consultation with a general practitioner, while patients in the control group completed their questionnaires in the waiting room after the consultation. After completion, the questionnaire was returned to the nurse manning the reception.

Registered sick leave data were retrieved from a national database named MiDAS hosted by the Swedish Social Insurance Agency, a government agency that provides financial security in the event of illness, in January 31, 2018. In Sweden, sick pay is paid by the employer for up to two weeks with one qualifying day. Thereafter, sickness benefits are handled by the Swedish Social Insurance Agency. Therefore, the data from the Social Insurance Agency only include sick leave from day 15 and onwards of a sick leave spell.

### Work stress questionnaire, WSQ

The WSQ was chosen for this study, since it has a transactional perspective on occupation [21]. Thereby, the interdependence between personal characteristics and aspects of work and private life is considered to capture the self-assessed work-related stress [38]. The self-completion questionnaire was originally developed in a primary health care context to assist health care professionals in assessing patients’ risk for sick leave due to work-related stress irrespective of diagnosis as a complement to the regular diagnostic procedure. Since then, the WSQ has been used in several different study populations [23, 24, 37, 39]. The 21 items included concern both work-related factors, individual characteristics and the perceived stress thereof (Additional file 1). The items are categorized in four dimensions:

1. Influence at work (item 1–4) include both decision authority and consideration of opinions related to the conduct of work tasks and the work place in more general terms;
2. Indistinct organization and conflicts (item 5–11), concern the division of tasks, goals, and decision making as well as the prevalence and handling of conflicts caused by an indistinct organisation or due to other causes.
3. Individual demands and commitment (item 12–18); concern the individual’s perceived demands (self-imposed demands in relation to demands imposed by work) and the commitment to work as well as the effect thereof on setting limits, taking responsibility and hours worked.
4. Work to leisure time interference (item 19–21) include the effect that work has on time spent with nearest and friends as well as on recreational activities.

The design of the WSQ is based on both theory and empirical data. A more thorough description of the development and content of the WSQ can be found in two articles by Holmgren et al. [26, 38]. The face validity and the test–retest reliability has been tested for women and men separately and found satisfying [38, 39].

### Assessment of variables

#### Exposure

The exposure was assessed using data on self-assessed stress collected with the WSQ. The items (Additional file 1) were combined into six exposure variables, whereof the first four corresponded to WSQ dimensions:

1. *Influence at work* (dimension 1) included item 1–4 answered on a four-point scale with the alternatives “Yes, always,” “Yes, rather often,” “No, seldom” and “No, never.”
2. *Perceived stress due to indistinct organization and conflicts* (dimension 2) included item 5b-11b answered on a four-point scale with the alternatives “Not stressful,” “Less stressful,” “Stressful” and “Very stressful.”
3. *Perceived stress due to individual demands and commitment* (dimension 3) included item 12b-18b answered on a four-point scale with the alternatives “Not stressful,” “Less stressful,” “Stressful” and “Very stressful.”
4. *Work to leisure time interference* (dimension 4) included item 19–21 answered on a four-point scale with the alternatives “Yes, always,” “Yes, rather often,” “No, seldom” and “No, never.”
5. Number of dimensions indicating high exposure to stress.
6. The combined exposure from dimension 2 and dimension 3.

The median values for exposure variable 1–4 were calculated as described in the WSQ instructions (Additional file 1). The data was then dichotomized into high and low exposure to the different dimensions of work-related stress with the value 2 as a cut-off. For exposure variable 5, the dichotomized data from dimension 1-4 was transformed into three categories; 0, 1-2 and 3-4 dimensions contributing to stress-related ill health. For exposure variable 6, the dichotomized data from dimension 2 and 3 was transformed into three categories; low stress on both, low stress on one and high stress on both. Hence, having four dimensions indicating high exposure to stress meant ^1^seldom or never perceiving influence at work, ^2^perceiving work organization and occurring conflicts as stressful or very stressful, ^3^perceiving own work demands and commitment as stressful or very stressful, and ^4^always or rather often perceiving interference between work and leisure.

#### Outcome

The outcome was the number of registered gross sick-days during the study period, which was 12 months from baseline. The continuous sick-days variable was dichotomized using 15 days of sick leave as cut-off.

#### Background variables

Sex, age, education level, occupational class and marital status were included in the analysis. The selection was made from available data collected at baseline based on literature on work, stress and sick leave.

Sex was measured as nominal variable with two categories; woman and man.

Age was measured as a continuous variable, which was transformed into an ordinal variable with three age groups.

Educational level was measured as an ordinal variable with the alternatives elementary school not completed, elementary school, high school 2 years, high school 3–4 years, university less than 3 years and university 3 years or more. The categories were then summarized in three ordered categories; elementary school, high school and university.

Occupational class was measured as a nominal variable based on the respondent’s occupation. The data was then categorized according to the Swedish Socioeconomic Classification of persons in the labour force [40] and summarized in three categories; high-level non manual, medium/low non-manual and skilled/unskilled manual.

Marital status was measured as a nominal variable with three categories: married/cohabitant, single, and long-term relationship. The categories were collapsed into two categories; not single and single.

### Statistical analysis

Previous studies from the RCT have shown that sick leave was not affected by the intervention [41, 42]. In addition, the results from a Pearson's χ2 test showed that intervention group affiliation had no statistically significant effect on being on sick leave 15 days or more during the study period (p-value 0.57). All participants from the trial were therefore included in the present study, regardless of intervention group allocation.

The normality of the continuous outcome variable *number of registered gross sick-days 12 months after baseline* was tested with the Shapiro–Wilk test. The test results indicated that the data significantly differed from a normal distribution and therefore logistic regression was used to analyse the association between self-assessed work-related stress and days of sick leave.

Descriptive statistics were calculated for the demographic and socioeconomic variables as well as for the exposure and outcome variables. The main analysis included an estimation of odds ratios (OR) and 95% confidence intervals (95% CI) with bivariate logistic regression to evaluate the association between self-assessed work-related stress and registered sick leave. In addition, multivariable regression was used to control for several background variables. The selection of background variables for the analysis was based on significance testing of the association between each background variable and the outcome using Pearson’s χ^2^ test. A cut-off value for inclusion was set to a p-value below 0.25 for each variable [43]. The variables that fulfilled the criterion were added into the multivariate analysis one at a time and thereafter all together. If the odds ratio changed more than 10% between the unadjusted and the adjusted analysis, the variable added was considered to affect the association between work-related stress and sick leave (44, p. 223). A confidence interval for the odds ratio covering one was interpreted as a statistically insignificant association.

Potential multicollinearity between the background variables included in the logistic regression was tested by analyses of correlation. The variables were not exhibiting multicollinearity, since the correlation coefficients were all well below 0.8 and the VIF well below 2.5 for all the potential covariates [45].

All statistical analyses were performed using IBM SPSS Statistics for Windows, version 25.0.

## Results

### Sample characteristics

The study population included the 232 participants who had completed the WSQ. As seen in Table 1, 153 (66%) were women and 79 (34%) were men, 117 (50%) were between 31–50 years of age and 103 (44%) had a university degree. Altogether, 36% of the population were on sick leave for 15 days or more during the 12 months period after baseline.

**Table 1:** Association between registered sick leave and sociodemographic characteristics for the 232 participants

Variable		Total (*N* = 232)		Proportion with sick leave ≥ 15 days			
		n	(%)	Yes	%	*p*-value^1^	OR (95% CI)^a^
Total		232		83	36		
Sex						0.35	
	Male	79	34	25	32		1.00
	Female	153	66	58	38		1.32 (0.74;2.35)
Age (years)						0.58	
	18–30	41	18	14	34		1.00
	31–50	117	50	39	33		0.96 (0.46;2.04)
	51–64	74	32	30	41		1,32 (0.59;2.91)
Education level						**0.21**	
	University	103	44	32	31		1.00
	High school	106	46	40	38		1.35 (0.76;2.39)
	Elementary school	22	10	11	50		2.22 (0.87;5.65)
	Missing	1	0	0			
Occupational class						**0.16**	
	High-level non-manual	42	18	11	26		1.00
	Medium/low non-manual	100	43	34	34		1.45 (0.65;3.24)
	Skilled/unskilled manual	89	38	38	43		2.10 (0.94;4.70)
	Missing	1	0	0			
Marital status						**0.19**	
	Not single	184	80	63	34		1.00
	Single	45	20	20	44		1.55 (0.80;3.00)
	Missing	2	0	0			

^1^p-value, Pearson’s χ^2^ test for having or not having 15 days of sick leave or more. Values below 0.25 are marked with bold text as the corresponding variables then were treated as potential covariate to be included in the logistic regression analyses

^a^Odds ratio with 95% confidence interval, bivariate logistic regression

**Fig. 1:**
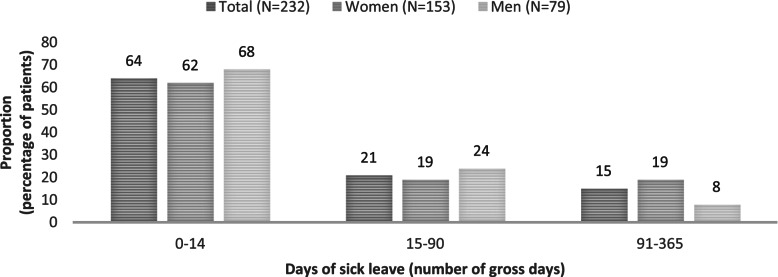
Distribution of sick leave during 12 months after baseline

Among women, 19% (*n* = 29) were on registered sick leave for 15–90 days and 19% (*n* = 29) were on registered sick leave for 91–365 days during the 12 months period (Fig. 1). For men, the corresponding values were 24% (*n* = 19) and 8% (*n* = 6). However, the gender difference was not statistically significant (p-value 0.07).

As seen in Table 2, 41% (*n* = 94) of the 232 participants perceived that they had low influence at work. In addition, 21% (*n* = 49) reported high stress due to indistinct organization and conflicts, while 45% (*n* = 105) reported high stress due to individual demands and commitment. Further, 40% (*n* = 93) perceived that they had high interference between work and leisure time. There were statistically significant differences between men and women concerning the perception of influence at work (p-value 0.048). The proportion perceiving low influence was higher among women than among men.

**Table 2:** Self-assessed work-related stressors and stress as measured with the Work Stress Questionnaire (WSQ)

Self-assessed work-related stressors and stress		Total (*N* = 232)		Women (*N* = 153)		Men (*N* = 79)		*p*-value^1^
		n	%	n	%	n	%	
Influence at work	High	138	59	84	55	54	68	0.048
	Low	94	41	69	45	25	32	
Stress due to indistinct organization and conflicts	Low	183	79	121	79	62	78	0.915
	High	49	21	32	21	17	22	
Stress due to individual demands and commitment	Low	127	55	78	51	49	62	0.109
	High	105	45	75	49	30	38	
Work interference with leisure time	Low	140	60	88	58	52	66	0.220
	High	93	40	65	42	27	34	

^1^Pearson's χ2 test

### Association between work-related stress and sick leave

Pearson’s χ2 test showed that educational level, occupational class and marital status could potentially affected the association between being on sick leave 15 days or more and perceiving high stress as measured with the WSQ, since the p-values for these associations were all below 0.25 (marked with bold type in Table 1).

All the WSQ variables were significantly associated with registered sick within 12 months after baseline (Table 3). The unadjusted odds were approximately twice as high for patients perceiving low influence at work (OR 2.07 95% CI 1.20;3.57), high stress due to indistinct organization and conflicts (OR 2.25 95% CI 1.18;4.26), high stress due to individual demands and commitment (OR 2.21 95% CI 1.28;3.82) or high interference between work and leisure time (OR 2.19 95% CI 1.27;3.80). Having a high effect from three or four of the WSQ dimensions quadrupled the odds of being on registered sick leave during the period, compared to having high effect from none of the dimensions. In addition, perceiving high stress due to both indistinct organization and conflicts as well as individual demands and commitment also quadrupled the odds of being on registered sick leave compared to having low on both. Further, adding the variables educational level, occupational class and marital status one by one in multivariate analyses only resulted in small changes in point estimates and confidence intervals. Neither did the three variables together result in any significant changes (Table 3). Hence, the analysis did not indicate that educational level, occupational class and marital status were important confounders for the association between work-related stress as measured with the WSQ and registered sick leave.

**Table 3:** Association between work-related stress measured with the Work Stress Questionnaire and registered sick leave (*N* = 232)

Variable		Sick leave ≥ 15 days		Unadjusted	Adjusted for educational level, occupational class and marital status
		Yes	%	OR (95% CI)	OR (95% CI)
Influence at work	High	40	17	1.00	1.00
	Low	43	19	2.07 (1.20:3.57)	2.00 (1.14;3.52)
Stress due to organization and conflicts	Low	58	25	1.00	1.00
	High	25	11	2.25 (1,18:4,26)	2.28 (1.17;4.44)
Stress due to demands and commitment	Low	35	15	1.00	1.00
	High	48	21	2,21 (1,28;3,82)	2.44 (1.37;4.33)
Work/leisure time interference	Low	40	17	1.00	1.00
	High	43	19	2,19 (1,27;3,80)	2.32 (1.31;4.11)
Effect from any dimension^a^	0 dim	15	6	1.00	1.00
	1–2 dim	36	16	1.97 (0.98;3.97)	1.90 (0.92;3.94)
	3–4 dim	32	14	4.35 (2.02;9,36)	4.56 (2.05;10.16)
Combination of perceived stress^b^	Low on both	30	13	1.00	1.00
	High on one	33	14	1.62 (0.89;2.96)	1.83 (0.97;3.46)
	High on both	20	9	4.15 (1.84;9.38)	4.54 (1.93;10.69)

^a^Low influence at work, high perceived stress due to indistinct organization and conflicts, high perceived stress due to individual demands and commitment or high work to leisure time interference

^b^Perceived stress due to indistinct organization and conflicts in combination with perceived stress due to individual demands and commitment

## Discussion

### Main findings

This study showed that one third of the participating primary health care patients were on registered sick leave, that is sick leave spells lasting more than 14 days, within a year after inclusion. In addition, there was a positive association between work-related stress and registered sick leave for patients seeking care at primary health care centres for mental and/or physical health complaints. Influence at work, indistinct organization and conflicts, individual demands and commitment as well as interference between work and leisure time were all important aspects associated with future sick leave, since each of the aspects more than doubled the odds of sick leave. Having several of the work-related stressors and perceiving stress thereof quadrupled the odds of future registered sick leave. Sex, age, educational level, occupational class and marital status did not affect the association between work-related stress and sick leave.

### Interpretation of findings

Findings from the study in hand indicate that work stressors and perceived stress thereof are common among primary health care patients seeking care for mental and/or physical health complaints. One fifth of the study population perceived high stress due to instinct organisation and conflicts and almost half of the population perceived high stress due to individual demands and commitment. The WSQ results from a prospective study among Swedish employed women seeking care at primary health care centres [24] showed that increased workload and difficulties setting limits were the most important stressors for the stress perceived. As a comparison, in a general population of Swedish employed women one in ten perceived high stress owing to indistinct organisation and conflicts, and one in four perceived high stress owing to individual demands and commitment as measured by the WSQ [23]. Moreover, a Swedish nationwide survey conducted in 2016 showed that approximately twenty percent of the Swedish population reported work-related stress [46]. These findings indicate that a higher proportion of primary health care patients with mental and/or physical health complaints report work-related stress compared to the general population. Thereby implying that these patients are more likely to have work-related stress and subsequent ill health compared to the general population. In agreement, approximately one third of the primary health care patents in the present study were on registered sick leave, independent of cause, within a year after baseline. This figure is three times as a high compared to the general population aged 18–64 during 2016 [47], which was the year the study was performed. A possible explanation for the differences in stress and sick leave compared to the general population is that people with work-related stress and ill health thereof consult primary health care to a higher extent than those who do not. On the other hand, people who suffer from and seek care for different types of ill health might also find it hard to cope at work and consequently perceive work-related stress and ill health.

Approximately forty percent of the study population perceived low influence at work, which include both decision authority and consideration of opinions. In comparison, this number is twice as high as what was found when the WSQ was used for the assessment of work-related stress in a general population of Swedish women [23]. Another finding from the study was that the odds of future sick leave doubled for patients perceiving low influence. The association confirms prior findings where the WSQ has been used to assess work-related stress [23, 24] as well as other studies using slightly different measures [13, 30, 48]. However, based on research findings, the interplay between influence at work and sick leave seems to be complex. Among French clerks and blue-collar workers, age and occupational group affected the association between work autonomy and sick leave [49]. In addition, among white-collar workers in Canada women, contrary to men, had an increased risk of sick leave when perceiving both high control and high demands at work compared to perceiving low on both measures [30]. Moreover, occupational group affiliation affected the strength of the association between influence at work and sick leave among Danes [22]. The inconclusive research findings could be explained by differences in study design and settings. However, the findings might also reflect the complex association between influence at work and other contextual aspects both within and outside of work. Nonetheless, sex, age, educational level, occupational class and marital status did not affect the association in the present study.

Work commitment, that is a frame of mind that binds an individual to a course of action of relevance to work [50], is seen as important constructs in organizational and health research [16, 51]. In accordance, this study showed that perceiving high stress due to demands and commitment doubled the risk of being on sick leave. A strong personal commitment to an organisation has been characterized as a willingness to exert high efforts for the organization, a desire to remain in the organization, and a belief in major goals and values of the organization [51] and therefore an important organisational and personal resource. Excessive work commitment has though been described as a personal characteristic and a risk factor for stress-related ill health [16, 52]. The thought of excessive commitment as being a personality trait has been questioned though, as commitment also can be a response pattern subjective to changes in the work environment [53]. Founded on system theory, Katz and Kahn [54] stated that the requirements, expectations and larger patterned behaviours of societies and organizations, to a considerable extent determine the role perception and the work performance of the employee. In this study work commitment was therefore viewed as more of a state open to change depending on the social and organisational situation at work rather than a stable individual characteristic.

The study in hand showed that perceiving stress due to an indistinct organisation with increased workload, unclear goals, ill-defined work tasks and/or having conflicts at work, increased the odds of future registered sick leave. Findings from a critical review on organisational climate and employee health outcomes [55], showed that poor organisational climate influence both the physical and psychological health of employees negatively. According to Loh et al. [55] the organisational climate acts as a social cue for what is considered as expected and favourable behaviours; behaviours that hopefully contribute to positive employee health outcomes. Possible moderators of organisational climate and health relationships are rarely investigated [55]. However, the results from a longitudinal study with employees of a forest industry corporation in Finland, Vännänen et al. [15] showed that blue-collar women, but not white-collar women or men, with a poor organizational climate had a significant risk of short-term sick leave. The authors therefore argued that actions to reduce organizational level psychosocial risk factors of sick leave have to match the specific needs of each socioeconomic group. Another notable finding from the present study was that perceiving high stress due to both indistinct organization and conflicts and high stress due to individual demands and work commitment quadrupled the odds of registered sick leave compared to perceiving low stress on both these measures. The result confirms previous finding from a general Swedish working population study [25] and a primary health care study [24] where a combination of poor organizational climate and high work commitment, as measured with the WSQ, increased the odds of future sick leave.

As described above, it is important to consider the individual's physiological, psychological and behavioural responses to stressors as well as the interaction between the individual and environment to understand the causes of work-related stress and subsequent ill health. Since primary health care is often the first health care contact for persons perceiving all types of ill health, understanding the underlying causes for the ill health and to diagnose are important parts of the work performed by the general practitioners. Their work is often founded on biomedical thinking and approaches, although the value and importance of other models have been acknowledged [56]. However, there are few ques guiding their work in this direction, since guidelines, indicators and diagnostic tools are primarily biomedically oriented [56]. To be able to identify, understand and treat the variety of expressions of ill health due to work-related stress, the work performed by the primary health care professionals has to be based on different views and perspectives, and tools like the WSQ could then be useful. Using other frameworks than the traditional exposure-disease framework in occupational health research and primary health care research may also nurture new types of research questions embedded in societal contexts [1].

### Strengths and weaknesses

A strength of this study is the longitudinal prospective study design, which made it possible to test the causal hypothesis for the study in hand, that is, that Swedish primary health care patients perceiving work-related stressors and stress, as measured with the WSQ, have increased odds of future registered sick leave.

The population of interest included working individuals, presently not on sick leave, seeking care at Swedish primary health care centres for mental and/or physical health complaints that might be stress-related. The inclusion criteria were carefully chosen, to obtain a selection of study participants where work-related stress most likely was prevalent and where there was a risk of sick leave. Since stress effects health in multiple ways, the symptoms that were included had to be wide-ranging in scope. The inclusion criteria were therefore considered as relevant to define the population at risk of sick leave due to work-related stress. Nonetheless, it is fair to question if the patients at the seven primary health care centres in western Sweden were representative for patients nationwide. However, the centres included were both private and public run as well as located both in urban and rural areas, which indicate an inclusion of patients with different backgrounds.

The present study as well as other research on psychosocial factors in occupational settings rely on self-report questionnaire measures of work characteristics [5, 14, 20, 22, 30, 31]. Using self-reports have though been criticized for not being objective, since the data might not truly reflect the individual’s assessment of stressors and stress [57]. However, using self-reports was considered relevant in the study, since it is the individual’s perception and experiences of the work environment that is of interest. In addition, when assessing the association between stress and sick leave the choice of questionnaire is important, to certify that the components of the working life that are critical for adverse health effects are included. Therefore, it cannot be ruled out that other psychosocial work factors than those included in the WSQ could be important.

Using sick leave as an outcome measure was considered relevant for this primary health care study, as prior research has shown an association between the psychosocial environment, stress and sick leave in different setting as well as in population-based studies [13–15, 25, 31]. Since registered sick leave was used as an outcome measure, spells shorter than fifteen days were not included in the analyses. However, the association between ill health and sick leave is according to Marmot et al. [58] stronger for longer than for shorter spells. It is therefore reasonable to assume that registered sick leave is a useful measure of the ill health perceived. In addition, using registered sick leave instead of self-reported sick leave prevents recall bias. Moreover, stress can cause mental, physical and behavioural ill health [9–11] and the incidence and recurrence of sick leave depends on the sick leave diagnosis [59]. Therefore, if diagnosis-specific sick leave rather than all-cause sick leave would have been used, the understanding of the association between stress and sick leave could have increased.

Logistic regression was used for the analysis, due to the skewness of the sick leave data. In addition, the method is frequently used in etiological analyses of sick leave data [25, 31, 48]. Covariates known to affect the association between self-assessed work-related stress and registered sick leave were included in the analyses [12]. However, a concern when defining the sick day variable was how to dichotomize the continuous outcome variable. In Sweden, sickness benefits for the first 14 days of sick leave is paid by the employer, and is therefore not recorded in the social insurance registers. Fifteen days of sick leave was therefore chosen as a cut-off point to reflect the legislation, but choosing a different cut-off point could affect the results. The analysis did not show that gender had an effect on the association between stress and sick leave, but performing stratified analyses could have detected differences in covariates and confounders between strata.

## Conclusion

Work stressors and perceived stress thereof were prevalent among the working age primary health care patients seeking care for mental and/or physical health complaints. In addition, one third of the study patients had 15 days of sick leave or more in one year. The study also showed that perceiving stressors and stress due to the organization, the specific work context, the individual characteristics and the interference between work and leisure time were all integral areas affecting future sick leave. Targeted measures directed to the prevention of stress-related ill health and especially work-related stress could therefore be useful in primary health care settings to improve the health of the working population and to reduce the number of sick days. In addition, a wide spectrum of factors has to be considered, to capture significant factors of the dynamic interaction between the individual and the work environment for the relationship between work-related stress and sick leave. Further research is needed to address specific subgroups based on for instance gender or occupational sectors and to account for long-term health effects of stress, but also to study the effect that the reason for consultation and the sick leave diagnosis could have on the relationship.

## Supplementary Information

**Additionalfile 1.** Microsoft Word Document (.docx). The Work StressQuestionnaire including instructions for evaluation.

## Data Availability

The study was based on data collected for this study specifically as well as register data that were retrieved from a national database hosted by the Swedish Social Insurance Agency. Due to restrictions in the ethical approval and restrictions applied to the availability of the register data, the datasets generated and analysed during the study are not publicly available. However, the data are available from the corresponding author upon reasonable request.

## Abbreviations

CI: Confidence Interval
OR: Odds Ratio
WSQ: Work Stress Questionnaire
