# Supplementary material for: Work-related stress and future sick leave in a working population seeking care at primary health care centres: a prospective longitudinal study using the WSQ
Source: BMC Public Health. 2022 Apr 28;22:851. doi: 10.1186/s12889-022-13269-8 (PMC9047382; doi:10.1186/s12889-022-13269-8)
Supplement: Supplementary file 1 — Additionalfile 1. Microsoft Word Document (.docx). The Work StressQuestionnaire including instructions for evaluation. [file 12889_2022_13269_MOESM1_ESM.docx]

**The Work Stress Questionnaire including instructions for evaluation**

The Work Stress Questionnaire (WSQ) is a self-assessment questionnaire measuring perceived work-related stress. The 21 questions are grouped in four dimensions:

1. ***Influence at work****, including four items that can be answered Yes, always, Yes, often, No, rarely or No, never.*
2. ***Indistinct organization and conflicts***, *including seven items that can be answered Yes, Partly, or No.*
3. ***Individual demands and commitment***, *including seven items that can be answered Yes, Partly or No.*
4. ***Work to leisure time interference***, including three items that can be answered Yes, always, Yes, often, No, rarely or No, never.

Each question in the dimensions *Indistinct organization and conflicts* and *individual demands and commitment* has a supplementary question ”Do you perceive that as stressful?” that is answered Not stressful, Less stressful, Stressful or Very stressful. Thereby, two additional dimensions are formed: ***Perceived stress due to indistinct organization and conflicts*** (seven items) and ***Perceived stress due to individual demands and commitment*** (seven items)*.*

The perceived work-related stress is calculated by using the values behind each answer in the questionnaire, see below. The median is calculated for four of the dimensions:

- Influence at work, item 1-4.
- Perceived stress due to indistinct organization and conflicts, item 5b-11b.
- Perceived stress due to individual demands and commitment, item 12b-18b.
- Work to leisure time interference, item 19-21.

The median is the central number of the values when they are sorted from smallest to largest. When the median is calculated for an even number, the higher of the two in the middle is used.

| **Dimension** | **Sorted numbers** | | | | | | | | | | | | **Median** |
| --- | --- | --- | --- | --- | --- | --- | --- | --- | --- | --- | --- | --- | --- |
| Influence at work (four items) |  | |  | | | |  | | | |  | |  |
| Perceived stress due to indistinct organization and conflicts (seven items) |  |  | |  | |  | |  | |  | |  |  |
| Perceived stress due to individual demands and commitment (seven items) |  |  | |  | |  | |  | |  | |  |  |
| Work to leisure time interference (three items) |  | | | |  | | | |  | | | |  |

Literature:

- Frantz A, Holmgren K. The Work Stress Questionnaire (WSQ) - Reliability and face validity among male workers. BMC Public Health, 2019, Vol 19, Iss 1. 2019;19(1).
- Holmgren K, Fjällström-Lundgren M, Hensing G. Early identification of work-related stress predicted sickness absence in employed women with musculoskeletal or mental disorders: a prospective, longitudinal study in a primary health care setting. Disabil Rehabil. 2013;35(5):418-426.
- Holmgren K, Dahlin-Ivanoff S, Björkelund C, et al. The prevalence of work-related stress, and its association with self-perceived health and sick-leave, in a population of employed Swedish women. BMC Public Health. 2009;9.
- Holmgren K, Hensing G, Dahlin-Ivanoff S. Development of a questionnaire assessing work-related stress in women - Identifying individuals who risk being put on sick leave. Disabil Rehabil. 2009;31(4):284-292.
- Holmgren K, Dahlin Ivanoff S. Women on sickness absence--views of possibilities and obstacles for returning to work. A focus group study. Disabil Rehabil. 2004 Feb 18;26(4):213-22.

Homepage: www.gu.se/forskning/tidas

| 01 Do you have time to finish your assignments? | □ yes, always | 1 |
| --- | --- | --- |
|  | □ yes, rather often | 2 |
|  | □ no, seldom | 3 |
|  | □ no, never | 4 |
| 02 Do you have the possibility to influence decisions at work? | □ yes, always | 1 |
|  | □ yes, rather often | 2 |
|  | □ no, seldom | 3 |
|  | □ no, never | 4 |
| 03 Does you supervisor consider your views? | □ yes, always | 1 |
|  | □ yes, rather often | 2 |
|  | □ no, seldom | 3 |
|  | □ no, never | 4 |
| 04 Can you decide on your work pace? | □ yes, always | 1 |
|  | □ yes, rather often | 2 |
|  | □ no, seldom | 3 |
|  | □ no, never | 4 |
| 05a Has your workload increased? | □ yes | 0 |
|  | □ no – if no, go to question **06a** | 1 |
| 05b If yes: Do you perceive that as stressful? | □ not stressful | 1 |
|  | □ less stressful | 2 |
|  | □ stressful | 3 |
|  | □ very stressful | 4 |
| 06a Are the goals for your workplace clear? | □ yes – if yes, continue to question **07a** | 1 |
|  | □ partly | 0 |
|  | □ no | 0 |
| 06b If partly or no: Do you perceive that as stressful? | □ not stressful | 1 |
|  | □ less stressful | 2 |
|  | □ stressful | 3 |
|  | □ very stressful | 4 |
| 07a Do you know which assignments your work tasks include? | □ yes – if yes, continue to question **08a** | 1 |
|  | □ partly | 0 |
|  | □ no | 0 |
| 07b If partly or no: Do you perceive that as stressful? | □ not stressful | 1 |
|  | □ less stressful | 2 |
|  | □ stressful | 3 |
|  | □ very stressful | 4 |
| 08a Do you know who is making decisions concerning your workplace? | □ yes – if yes, continue to question **09a** | 1 |
|  | □ partly | 0 |
|  | □ no | 0 |
| 08b If partly or no: Do you perceive that as stressful? | □ not stressful | 1 |
|  | □ less stressful | 2 |
|  | □ stressful | 3 |
|  | □ very stressful | 4 |
| 09a Are there any conflicts at work? | □ yes | 0 |
|  | □ no – if no, continue to question **12a** | 1 |
| 09b If yes: Do you perceive that as stressful? | □ not stressful | 1 |
|  | □ less stressful | 2 |
|  | □ stressful | 3 |
|  | □ very stressful | 4 |
| 10a Are you involved in any conflicts at your workplace? | □ yes | 0 |
|  | □ no – if no, continue to question **11a** | 1 |
| 10b If yes: Do you perceive that as stressful? | □ not stressful | 1 |
|  | □ less stressful | 2 |
|  | □ stressful | 3 |
|  | □ very stressful | 4 |
| 11a Has your supervisor done anything to solve the conflicts? | □ yes – if yes, continue to question **12a** | 1 |
|  | □ partly | 0 |
|  | □ no | 0 |
| 11b If partly or no: Do you perceive that as stressful? | □ not stressful | 1 |
|  | □ less stressful | 2 |
|  | □ stressful | 3 |
|  | □ very stressful | 4 |
| 12a Do you put high demands on yourself at work? | □ yes | 0 |
|  | □ no – if no, continue to question **13a** | 1 |
| 12b If yes: Do you perceive that as stressful? | □ not stressful | 1 |
|  | □ less stressful | 2 |
|  | □ stressful | 3 |
|  | □ very stressful | 4 |
| 13a Do you often get engaged in your work? | □ yes | 0 |
|  | □ no – if no, continue to question **14a** | 1 |
| 13b If yes: Do you perceive that as stressful? | □ not stressful | 1 |
|  | □ less stressful | 2 |
|  | □ stressful | 3 |
|  | □ very stressful | 4 |
| 14a Do you think about work after your working-day? | □ yes | 0 |
|  | □ partly | 0 |
|  | □ no – if no, continue to question **15a** | 1 |
| 14b If yes or partly: Do you perceive that as stressful? | □ not stressful | 1 |
|  | □ less stressful | 2 |
|  | □ stressful | 3 |
|  | □ very stressful | 4 |
| 15a Do you find it hard to set a limit to work assignment although you have a lot to do? | □ yes | 0 |
|  | □ partly | 0 |
|  | □ no – if no, continue to question **16a** | 1 |
| 15b If yes or partly: Do you perceive that as stressful? | □ not stressful | 1 |
|  | □ less stressful | 2 |
|  | □ stressful | 3 |
|  | □ very stressful | 4 |
| 16a Do you take more responsibility at work than you ought to? | □ yes | 0 |
|  | □ no – if no, continue to question **17a** | 1 |
| 16b If yes: Do you perceive that as stressful? | □ not stressful | 1 |
|  | □ less stressful | 2 |
|  | □ stressful | 3 |
|  | □ very stressful | 4 |
| 17a Do you work after ordinary working hours to finish your assignments? | □ yes | 0 |
|  | □ partly | 0 |
|  | □ no – if no, continue to question **18a** | 1 |
| 17b If yes or partly: Do you perceive that as stressful? | □ not stressful | 1 |
|  | □ less stressful | 2 |
|  | □ stressful | 3 |
|  | □ very stressful | 4 |
| 18a Do you find it hard to sleep because your mind is occupied with work? | □ yes | 0 |
|  | □ partly | 0 |
|  | □ no – if no, continue to question **19** | 1 |
| 18b If yes or partly: Do you perceive that as stressful? | □ not stressful | 1 |
|  | □ less stressful | 2 |
|  | □ stressful | 3 |
|  | □ very stressful | 4 |
| 19 Due to work, do you find it hard to find time to be with your nearest? | □ yes, always | 4 |
|  | □ yes, rather often | 3 |
|  | □ no, seldom | 2 |
|  | □ no, never | 1 |
| 20 Due to work, do you find it hard to find time to be with your friends? | □ yes, always | 4 |
|  | □ yes, rather often | 3 |
|  | □ no, seldom | 2 |
|  | □ no, never | 1 |
| 21 Due to work, do you find it hard to find time for your recreational activities? | □ yes, always | 4 |
|  | □ yes, rather often | 3 |
|  | □ no, seldom | 2 |
|  | □ no, never | 1 |
